# The Effect of Piroxicam on the Formation of Postoperative, Intraabdominal Adhesion in Rats

**DOI:** 10.4103/1319-3767.43276

**Published:** 2008-10

**Authors:** Hemmat Maghsoudi, Behnam Askary

**Affiliations:** Department of Surgery, Faculty of Medicine, University of Medical Sciences of Tabriz, Iran

**Keywords:** Peritoneal adhesions, piroxicam, prevention

## Abstract

**Background/Aims:**

Peritoneal adhesions are fibrous bands of tissues formed between organs that are normally separated and/or between organs and the internal body wall after peritoneal injury. Antiinflammatory agents were used to reduce the initial inflammatory response to tissue injury and, hence, the subsequent formation of adhesion. The aim of this study was to investigate the effect of intraperitoneal instillation of piroxicam on intraperitoneal adhesions.

**Methods:**

Eighty Wistar rats were subjected to standardized lesion by using the scraping model and were randomly divided into four groups. Group I (control) received no treatment; groups II, III, and IV received 10-12.5 mL of 0.05, 0.1, and 0.2 mg/mL piroxicam solution, respectively, after surgery. On the 14th postoperative day, the adhesion intensity score, inflammatory cell reaction, and the number of adhesion bands were determined.

**Results:**

There were no rats with grade 0 adhesions in the control group. There were 10 rats (50%) with grade 2 and eight rats (40%) with grade 3 adhesions. The adhesion intensity (*P* < 0.0001) and the number of adhesion bands (*P* < 0.001) were significantly lower in groups III and IV. No significant difference was observed in the adhesion intensity or the number of adhesion bands between groups I and II.

**Conclusions:**

Intraperitoneal instillation of piroxicam solution might be useful for preventing peritoneal adhesions.

Adhesion formation after surgery is a significant cause of morbidity.[1,2] The formation of peritoneal adhesions is a specific peritoneal response to injury, which activates the cascades that leads to adhesion formation.[3] The inflammatory response, normally induced by infection or tissue injury, is crucial in controlling and eliminating infectious agents, as well as in promoting wound healing.[4] It is known that wound healing and adhesion formation have similar pathways, following the sequence of tissue inflammation, fibrin deposition, fibrin organization, collagen formation, and maturation.[5,6] Peritoneal adhesions are defined as pathological fibrin bands that develop between any surfaces in the peritoneal cavity. For the development of adhesions between two surfaces inside the peritoneum, there must be peritoneal mesothelial damage on at least one surface.[7] A fibrinous exudate is released a few hours after mesothelial damage. When the exudate is absorbed, fibrous bands and newly formed capillary vessels remain at the site of surgery, and these structures form the permanent fibrotic adhesion.[7,8]

Postoperative peritoneal adhesions (PPA) develop after 90% of all laparotomies.[9,10] Intestinal obstruction related to PPA develops in 1% of all surgical interventions and in 3% of all laparotomies.[9] PPA is the cause of 15–20% of all female infertility cases.[11,12] Enterocutaneous fistuli, intraabdominal abscesses, ureteral obstruction, and chronic abdominal pain may also develop as a result of PPA.[13] Several agents have been used to solve this very serious complication of abdominal surgery.[14,15] Much research is being conducted by biomedical companies in this regard, and there are also several barrier products available in the market.[15,16]

Many methods and substances such as sodium citrate, heparin, dextran, prostigmine, olive oil, steroids, and antihistamines have been used, either locally or systemically, in an effort to reduce or prevent PPA formation.[14]

Mechanical separation of the peritoneal surfaces has used either organic (ox peritoneum) or bioabsorbable inorganic (Seprafilm1 Genzyme Co., USA) membranes.[16] However, unfortunately, these materials and procedures have only had limited success in abdominal surgery.

In this study, we planned to investigate the effect of piroxicam in preventing the development of PPA after the wound-healing process in rats.

## MATERIALS AND METHODS

The study was conducted in the Experimental Animal Raising and Research Laboratory, Tabriz University of Medical Sciences. After obtaining approval from the local ethics committee, eighty 6-month-old, outbred, male Wistar rats weighing 200–250 g were obtained from the aforementioned laboratory for the study. The rats were kept in air-conditioned colony rooms and given standard rat chow diet and water ad libitum. After overnight fasting, all animals were anesthetized with 25 mg/kg ketamine and 4 mg/kg xylazine. The abdomen was preoperatively shaved and swabbed with a povidine iodine solution. To remove powder particles, operation gloves were washed thoroughly with saline. The same researcher performed all surgical procedures. A 5-cm midline incision was made, and the abdomen was opened under clean surgical conditions. The terminal ileum and cecum of all animals were mobilized and placed on a wet gauze. Both sides of a 10-cm terminal ileum segment just proximal to the cecum as well as the cecum were scraped until there were serosal petechiae on the intestinal surfaces. Later, the arteries of the scraped segments were clamped for 1 minute to induce transient ischemia (the scraping model).[21]

To eliminate any possible differences between the rats, the animals were randomly assigned to four groups by using a number random table and treated as follows: group I (n = 20) was the control group and received no treatment; groups II, III, and IV (n = 20 per group) received intraperitoneal instillation of 10–12.5 mL of 0.05, 0.1, and 0.2 mg/mL piroxicam solution, respectively,. The abdominal incision was then closed in two layers with 3-0 propylene suture. The animals were then given the regular pellet (state manufacturer's) food. All rats were killed on the 14^th^ day after being anesthetized with an overdose of ethyl ether before re-laparotomy. Two observers, who were blinded to treatment groups, assessed the extent of adhesion formation. The frequency and intensity of adhesions were recorded, and the mean of the two observers' scores was used for statistical analysis. Adhesions were graded as 0 to 3 based on their severity[22] [Table 1].

**Table 1:** Adhesion grading according to Evans model[22]

Grade	Grading of adhesions
0	No adhesions
1	Spontaneously separating adhesions
2	Adhesions separating by traction
3	Adhesions separating by dissection

The injured terminal walls of the ileum and cecum with fibrous adhesions were excised to confirm the scraping of the walls as well as the formation of adhesion. The specimens were fixed in 70% alcohol, dehydrated, and embedded in paraffin wax. Sections were cut at a thickness of 5 mm and stained with hematoxylin and eosin (H & E).

The Mann–Whitney U test was used as a nonparametric test to determine the differences in adhesion grading based on severity. A chi-square test was used to analyze the number of adhesion bands between the different groups. Significance was set at ***P*** < 0.05, and ***P*** < 0.001 was considered highly significant.

## RESULTS

The grading of adhesions in each group is summarized in Table 2. There were no animals with grade 0 in the control group but two in grade 1. Group II contained three (15%) in grade 0 and two (10%) in grade 1. Groups III and IV contained eight (40%) rats with grade 0, eight rats (40%) with grade 1, And four (20%) rats with grade 2, but no rats were with grade 3. A comparison of groups by Mann–Whitney U test indicated that the decrease in severity of adhesion bands was highly significant in groups III and IV (***P*** < 0.001). In group 1, all the animals had adhesion bands, whereas three rats in group II and eight rats each in groups III and IV had no adhesion bands. A comparison of groups indicated that the decrease in the number of adhesion bands was highly significant in groups III and IV (***P*** < 0.001). The number of adhesion bands in each group is summarized in Table 3. Nonspecific inflammatory changes were seen in the damaged cecal and terminal ileal walls in all specimens. The dominant inflammatory cells were polymorphonuclear neutrophils. The inflammation was less severe in groups III and IV, and the dominant cells were macrophage and polymorphonuclear neutrophils and were fewer in number than those found in the control group.

**Table 2:** Adhesion grading of the groups

Grade	Group I	Group II	Group III	Group IV
0	0	3	8	8
1	2	2	8	8
2	10	9	4	4
3	8	6	0	0
Total	20	20	20	20

*P* < 0.001 between groups III, IV and group I according to the Mann-Whitney U-test

**Table 3:** Number of adhesion bands according to the number of cases in the groups

Number of adhesion bands	Group I	Group II	Group III	Group IV
0	0	3	8	8
1	4	9	8	7
2	7	6	4	4
3	5	1	0	1
4	3	0	0	0
5	0	1	0	0
6	1	0	0	0
Total	20	20	20	20

*P* < 0.001 between groups III, IV, and group I

## DISCUSSION

There are two major strategies for the prevention or reduction of adhesion. Surgical trauma is minimized within the peritoneum by careful handling of tissue, avoiding desiccation and ischemia, and by the sparing use of cautery, lasers, and retractors. Fewer adhesions form with laparoscopic surgical techniques because of reduced tissue trauma. The second major advance in the prevention of adhesion has been the introduction of barrier membranes and gels, which separate and create barriers between the damaged surfaces, allowing for adhesion-free healing. Modified, oxidized, regenerated cellulose and hyaluronic acid membranes or solutions have been shown to reduce adhesions in gynecological patients and are being investigated for their ability to prevent adhesion formation in general surgical patients.[23,24]

Antiinflammatory agents were used to reduce the initial inflammatory response to tissue injury and also the formation of any subsequent adhesion. Most animal studies have shown the effectiveness of nonsteroidal antiinflammatory drugs (NSAIDs) in the prevention of adhesions.[17,18] Despite that, other studies have failed to prove any beneficial effect of intramuscular or intraperitoneal administration of ibuprofen in the reduction of peritoneal adhesions in rat and rabbit models.[19,20] Unfortunately, no clinical trials with NSAIDs have been published to date, although several have been conducted.

In recommended doses, piroxicam seems to be the equivalent of aspirin, indomethacin, or naproxen for long-term treatment of rheumatoid arthritis or osteoarthritis. It is tolerated better than aspirin or indomethacin and, thus far, seems to be equivalent to its propionic acid derivatives in this regard. The principal advantage of piroxicam is its long-lasting activity, which permits the administration of a single daily dose. As with other NSAIDs, piroxicam can cause gastric erosions, and it prolongs the bleeding time. The reported incidence of adverse effects in patients who take piroxicam has ranged from 11 to 46%, and 4–12% of patients stopped using the drug because of side effects. Gastrointestinal reactions are the most common, but only < 5% of patients discontinue treatment for this reason; the incidence of peptic ulcer is < 1%. As do other NSAIDs, piroxicam alters the function of platelets, and it should be assumed that piroxicam will precipitate bronchoconstriction in those patients who are hypersensitive to aspirin. The usual daily dose for the relief of the signs and symptoms of rheumatoid arthritis or osteoarthritis is 20 mg; if desired, this may be given in two portions. Satisfactory responses can be achieved with piroxicam with plasma concentrations higher than 5–6 *µ*g/mL.[32]

There are many experimental models for the cause of peritoneal adhesions: the damaged uterine horn model, the ileal transection model, the large bowel anastomosis model, the peritoneal damage model, the bacterial peritonitis model, and the scraping model.[9,25–29]

The scraping model is very effective in causing peritoneal adhesions because there are two stages in the damage: direct mechanical intestinal wall damage from gauze scraping until petechial points appear and ischemic damage that is secondary to vascular clamping. We have chosen the scraping model for our study, as it mimics the abdominal surgery.

Several researchers have used different criteria for grading adhesions.[26,28,30,31] The Evans model grades adhesions as 0 to 3 based on their severity.[16] We have adopted the Evans model in this study because of its simplicity and rationale. A large number of studies have described the agents used to prevent the formation and reformation of peritoneal adhesions. Other antiinflammatory agents have been evaluated for this purpose, but piroxicam has never been tried in the prevention of PPA.[2]

We wanted to test the effect of piroxicam on PPA because of its antiinflammatory effect. NSAIDs fall into two classes: (i) carboxylic acids such as ibuprofen, indomethacin, naproxen, diflunisal, fenoprofen, and tolmetin and (ii) enolic acids including phenylbutazone and piroxicam. As a result of the ability of NSAIDs to block the enzymatic activity of cyclooxygenase, and therefore, the production of prostaglandins, prostacyclin, and thromboxanes, they have analgesic, antiinflammatory, and antipyretic properties.[32,33] Piroxicam is an effective antiinflammatory agent; it is about equal to indomethacin in potency as an inhibitor of prostaglandin biosynthesis in vitro. Piroxicam also exerts antipyretic and analgesic effects in experimental animals and humans. Piroxicam is approved in the United States for the treatment of rheumatoid arthritis and osteoarthritis. It has also been used in the treatment of ankylosing spondylitis, acute musculoskeletal disorders, and acute gout.[32,33]

In this study, we observed that the intraperitoneal instillation of 10–12.5 mL of 0.1 or 0.2 mg/mL piroxicam solution significantly decreases the development of PPA. In agreement with other studies, we showed that intraperitoneal instillation of piroxicam can inhibit inflammation and alter the intensity and frequency of the formation of adhesions in a concentration-dependent manner.[34]

In conclusion, this study suggests that the intraperitoneal instillation of piroxicam solution of concentrations higher than 0.1 mg/mL can decrease the formation of peritoneal adhesions.
